# Dehydroevodiamine Alleviates Ulcerative Colitis by Inhibiting the PI3K/AKT/NF-κB Signaling Pathway via Targeting AKT1 and Regulating Gut Microbes and Serum Metabolism

**DOI:** 10.3390/molecules29174031

**Published:** 2024-08-26

**Authors:** Xiao Ma, Qichao Hu, Tao Jiang, Yuan Chen, Wenwen Zhang, Pan Gao, Jinhao Zeng, Thomas Efferth

**Affiliations:** 1 State Key Laboratory of Southwestern Chinese Medicine Resources, School of Pharmacy, Chengdu University of Traditional Chinese Medicine, Chengdu 611137, China; huqichaotcm@stu.cdutcm.edu.cn (Q.H.); jt3194842831@163.com (T.J.); tochenyuan@163.com (Y.C.); zhangwenwen0211@163.com (W.Z.); lbyisgp427@126.com (P.G.); 2 TCM Regulating Metabolic Diseases Key Laboratory of Sichuan Province, Hospital of Chengdu University of Traditional Chinese Medicine, Chengdu 610072, China; 3 Department of Gastroenterology, Hospital of Chengdu University of Traditional Chinese Medicine, Chengdu 610072, China; 4 Department of Pharmaceutical Biology, Institute of Pharmaceutical and Biomedical Sciences, Johannes Gutenberg University, 55128 Mainz, Germany

**Keywords:** AKT1 inhibitor, metabolomics, phytotherapy, signal transduction, small molecular inhibitor, transcriptomics, ulcerative colitis

## Abstract

Ulcerative colitis (UC) is a typical inflammatory bowel disease (IBD), impairing the quality of life of patients. Dehydroevodiamine (DHE) is an active alkaloid isolated from *Tetradium ruticarpum* that exerts significant anti-inflammatory effects in gastrointestinal diseases. However, the effect and mechanisms of DHE on UC remain unclear. We performed a DSS-induced experimental UC rat model to reveal the efficacy and potential mechanisms of DHE on UC. HE and AB-PAS staining were used for the evaluation of pathologies, and 16S rRNA sequencing was used to detect changes in gut microbes. Metabolomics was used to detect changes in serum metabolites. Network pharmacology and transcriptomics were conducted to reveal the underlying mechanisms of DHE for UC. HuProt proteome microarrays, molecular docking, and SPR were used to reveal the targets of action of DHE. WB, RT-qPCR, and IHC were used to assess the action effects of DHE. DHE demonstrated significant alleviation of DSS-induced colitis symptoms in rats by suppressing inflammatory and oxidative stress responses, amending colonic barrier injury, and inhibiting apoptosis. In terms of gut microbial modulation, DHE decreased the abundance of *Allobaculum*, *Clostridium*, *Escherichia*, *Enterococcus*, and *Barnesiella* and increased the abundance of *Lactobacillus*, *Bifidobacterium*, and *SMB5*. Moreover, metabolomics suggested that the regulation of DHE in DSS-induced UC rats mainly involved aminoacyl-tRNA biosynthesis, vitamin B6 metabolism, phenylalanine, tyrosine, and so on. Mechanically, DHE alleviated UC in rats by targeting AKT1, thereby inhibiting the PI3K/AKT/NF-κB signaling pathway.

## 1. Introduction

Ulcerative colitis (UC), representing a typical chronic inflammatory bowel disease (IBD), is accompanied by clinical features, including diarrhea, abdominal pain, and mucopurulent and blood in stools, which severely reduces the quality of life [1,2]. The global incidence of UC is on the rise year by year [3]. Multifaceted causative factors contribute to the development of UC, such as genetic predisposition, immune imbalance, defective intestinal epithelial barrier damage, and environmental factors of life [4,5,6]. Currently, the management of UC aims to elevate the overall quality of life of patients by relieving symptoms as well as promoting the repair of intestinal epithelial barrier damage [7,8]. Concretely, the suppression of active inflammation and repair of damaged intestinal mucosa is an essential link in the management of UC [9,10]. Of the medicinal remedies for UC, 5-aminosalicylic acid (5-ASA) and salazosulfapyridine (SASP) are currently recommended clinically as first-line therapeutic agents for UC [11]. If the therapeutic effect of these medicinal interventions is insufficient, some complementary therapeutic modalities, such as glucocorticoids and immunosuppressants, are incorporated [12]. However, some non-negligible side effects are highlighted in clinical use, such as the risk of renal injury with prolonged administration of 5-ASA, which poses an obstacle to the long-term management and prognosis of UC [13]. Therefore, the discovery of new anti-UC drugs is essential [13].

Dehydroevodiamine (DHE) is a bioactive alkaloid isolated from *Tetradium ruticarpum* that exerts significant anti-inflammatory effects in diverse disorders. Initially, the anti-inflammatory effects of DHE were described as a resistance to lipopolysaccharide (LPS)-mediated expression of iNOS and COX-2 by downregulating the NF-κB signaling pathway [14]. In recent years, diverse investigations have revealed its protective role in various diseases. In central nervous system (CNS) disorders, DHE exerted neuroprotective effects in traumatic brain-injured mice by modeling the SIRT1/FOXO3a/Bim pathway [15]. The anti-inflammatory action of DHE has also contributed to its anti-tumor potential. DHE inhibits lung metastasis of colon cancer cells by promoting autophagy and inhibiting EMT [16]. More recently, DHE was found to be a novel DDIT3 activator that inhibits the development of pancreatic cancer by suppressing the AKT/mTOR signaling pathway [17]. Critically, our previous studies have focused on the ameliorative effects of DHE on acute and chronic gastritis. For example, DHE ameliorated chronic atrophic gastritis by inhibiting the HIF-1α/VEGF signaling pathway [18]. Meanwhile, DHE ameliorated acute gastritis by inhibiting the MAPK signaling pathway [19]. These results imply that DHE has a promising therapeutic role in the treatment of UC. However, no studies to date have focused on the potential of DHE for the treatment of UC. Based on these grounds, we performed a preliminary assessment of the therapeutic potential of DHE to ameliorate UC.

In this study, we found that DHE ameliorated DSS-induced UC in rats by improving the intestinal mucosal barrier, inhibiting apoptosis, and suppressing inflammation and oxidative stress. Meanwhile, DHE also alleviated UC by modulating alterations in serum metabolites and intestinal microbes. Mechanistically, we uncovered that DHE inhibited DSS-induced activation of the PI3K/AKT/NF-κB signaling pathway. In particular, our findings emphasize the role of DHE in directly targeting AKT1.

## 2. Results

### 2.1. DHE Relieved Symptoms of Colitis in UC Rats

**Figure 1:**
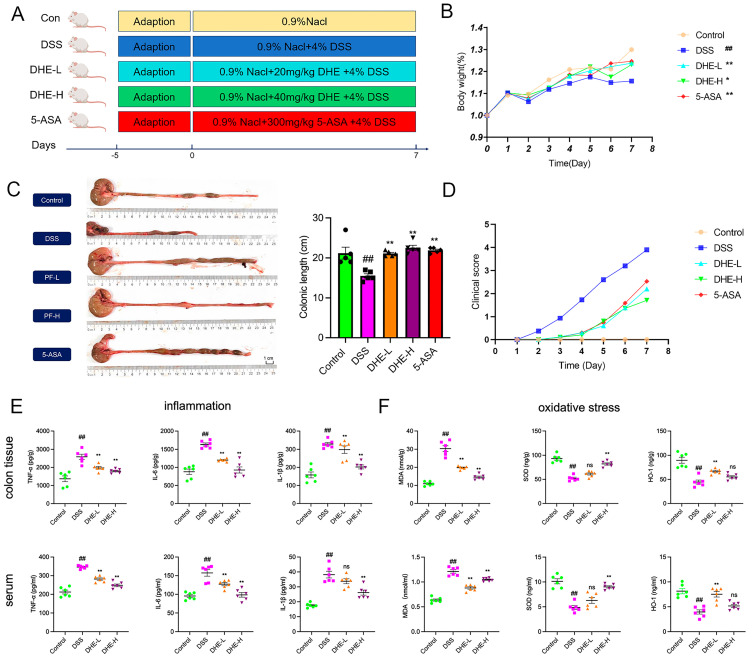
DHE reduced colitis symptoms, inflammation, and oxidative stress in rats. (**A**) UC modeling and drug administration process (*n* = 8). (**B**) The changes in body weights of rats (*n* = 8). (**C**) The changes and comparison of colonic length (*n* = 5). (**D**) The evaluation of clinical scores in each group (*n* = 8). (**E**) The level of IL-1β, IL-6, and TNF-α in colon tissues and serums (*n* = 6); Green symbols indicate the control; Pink symbols indicate the DSS; Yellow symbols indicate the DHE−L; Purple symbols indicate DHE−H. (**F**) The level of MDA, HO-1, and SOD in colon tissues and serums (*n* = 6); Green symbols indicate the control; Pink symbols indicate the DSS; Yellow symbols indicate the DHE−L; Purple symbols indicate DHE−H. All data are expressed as mean ± SEM (** *p* < 0.01 and * *p* < 0.05 indicate statistically significant differences from the DSS group; ^##^ *p* < 0.01 indicate statistically significant differences from the control group; ns, no significance).

Rats were continuously given 4% DSS for 7 days to induce the UC model. Starting from the day of modeling, varying doses of DHE and the positive control 5-ASA (300 mg/kg) were administered for 7 consecutive days (Figure 1A). DSS intake exacerbated the reduction in body weight of rats, while DHE and 5-ASA supplementation inhibited these changes (*p* < 0.01 or *p* < 0.05) (Figure 1B). The colon length of the DSS group was significantly reduced compared to other groups, while DHE and 5-ASA treatments effectively reversed these alterations (*p* < 0.01) (Figure 1C). Moreover, the reduction in disease activity index (DAI) score (shown as clinical score) indicated that DHE, as well as 5-ASA, notably decreased fecal blood and fecal viscosity in colitis rats (*p* < 0.01) (Figure 1D). These findings supported that treatment with DHE demonstrated a significant reduction of DSS-induced UC symptoms in rats.

### 2.2. DHE Reducing Inflammation and Oxidative Stress in UC Rats

Studies confirmed that the levels of pro-inflammatory factors such as IL-1β, IL-6, and TNF-α were significantly increased in UC rats [20]. To further evaluate the ameliorative effects of DHE in UC rats, we examined the indicators of inflammation and oxidative stress in rat intestinal tissues and serum. DSS intake significantly elevated intestinal and serum levels of IL-1β, IL-6, and TNF-α (*p* < 0.01), while DHE administration reduced these changes (*p* < 0.01) (Figure 1E). In addition, we assessed the effect of DHE in inhibiting DSS-induced oxidative stress in UC rats. DHE significantly reduced the DSS-induced rise in MDA and increased HO-1 and SOD levels in the intestines and serum of UC rats (*p* < 0.01) (Figure 1F). Collectively, DHE significantly suppressed inflammatory and oxidative stress responses in UC rats.

### 2.3. DHE Repaired the Intestinal Mucosal Barrier and Inhibited Apoptosis in UC Rats

**Figure 2:**
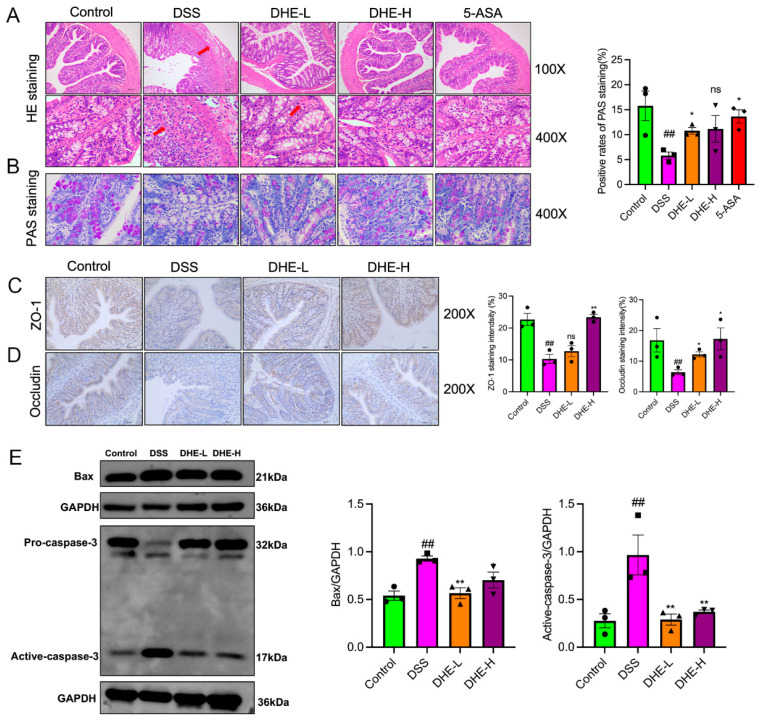
DHE repaired the intestinal mucosal barrier and inhibited apoptosis in UC rats. (**A**) HE staining (*n* = 3); The arrows indicate pathological changes. (**B**) AB-PAS staining (*n* = 3). (**C**) ZO-1 expression in colon tissue by IHC (*n* = 3). (**D**) Occludin expression in colon tissue by IHC (*n* = 3). (**E**) Bax and caspase-3 expressions by WB (*n* = 3). The dots in the statistical charts indicate individuals in each group. All data are expressed as mean ± SEM (** *p* < 0.01 and * *p* < 0.05 indicate statistically significant differences from the DSS group; ^##^ *p* < 0.01 indicate statistically significant differences from the control group; ns, no significance).

To reveal the effects of DHE on DSS-induced colonic impairment, we employed HE and PAS staining. The outcomes showed that compared to the control, the colonic tissues in the DSS group were relatively more damaged, as evidenced by lamellar necrosis of the mucosal layer, atrophy of the intestinal glands, disintegration of intestinal glandular epithelial cells, structural blurring, and reduction in the number of cupped cells accompanied by a higher number of inflammatory cell infiltration, while DHE and 5-ASA intakes decreased this trend (Figure 2A,B). ZO-1 and occludin are important tight junction proteins in the intestinal barrier and play a role in maintaining the intestinal barrier. Therefore, we examined the expression of ZO-1 and occludin in rat colon tissues using IHC. DSS significantly reduced the positive expression of ZO-1 and occludin compared with the control group (*p* < 0.01). Fortunately, DHE elevated the reduction of ZO-1 and occludin-positive expression after DSS intake (*p* < 0.01 or *p* < 0.05) (Figure 2C,D). In addition, we further examined the expression of pro-apoptotic proteins Bax and Caspase-3 in rat intestinal tissues using WB. DSS significantly promoted the expression of Bax and active-caspase-3 (*p* < 0.01), indicative of intestinal apoptosis, while DHE administration significantly inhibited this change (*p* < 0.01 or *p* < 0.05) (Figure 2E). Collectively, DHE amended colonic barrier injury in UC rats by enhancing the expression of intestinal tight junction proteins and inhibiting apoptosis.

### 2.4. DHE Mitigated Deregulation of Gut Microbes Induced by DSS in UC Rats

**Figure 3:**
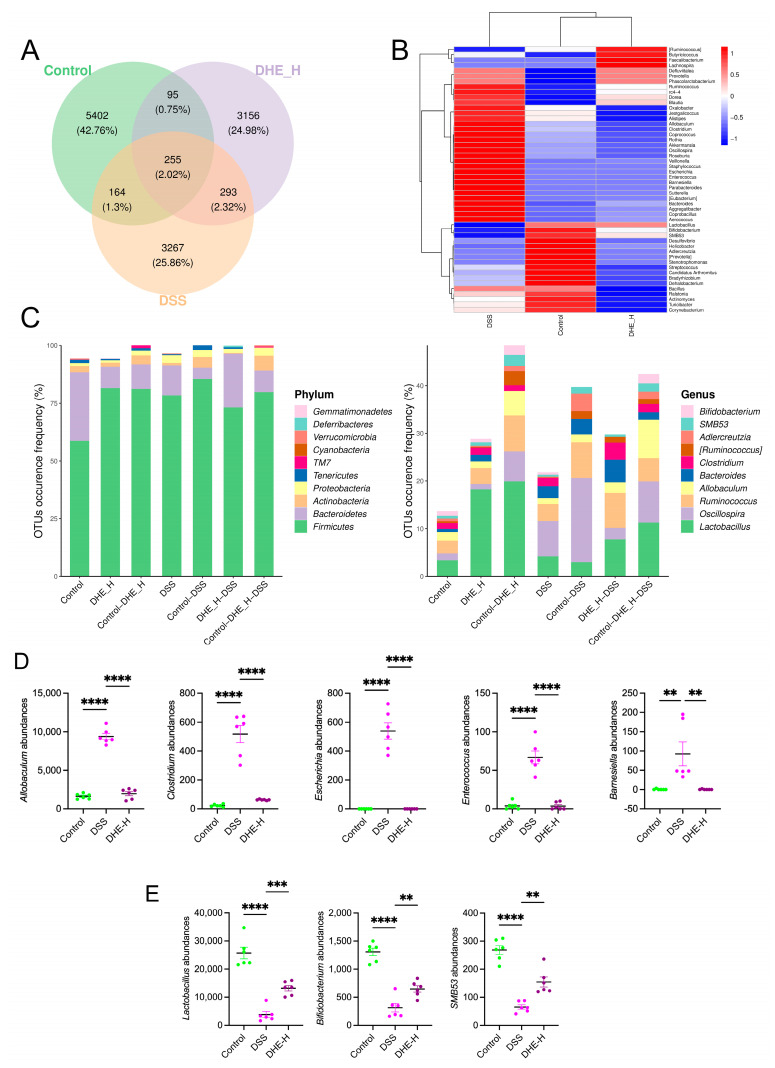
DHE mitigated deregulation of gut microbes induced by DSS in UC rats. (**A**) Venn diagram for gut microbes between the control group, the DSS group, and the DHE-H group. (**B**) The changes and comparison of gut microbes among the control group, the DSS group, and the DHE-H group. (**C**) OTU analysis. (**D**) DHE decreased the abundance of *Allobaculum*, *Clostridium*, *Escherichia*, *Enterococcus*, and *Barnesiella* (*n* = 6). (**E**) DHE intake increased the abundance of *Lactobacillus*, *Bifidobacterium*, and *SMB53* (*n* = 6). All data are expressed as mean ± SEM (** *p* < 0.01, *** *p* < 0.001 and **** *p* < 0.0001 indicate statistically significant differences).

The gut microbes play an influential role in maintaining homeostasis in intestinal tracts associated with UC [21]. To investigate the modulating role of DHE in alternations of gut microbes induced by DSS in rats, a 16S rRNA analysis was performed. The results showed the altered composition of rat intestinal microbes after DSS and DHE treatment compared with the control group (Figure 3A). In particular, DSS significantly exacerbated the imbalance of gut microbes, demonstrating an increase in most flora and a decrease in the abundance of some (Figure 3B,C). In detail, DSS significantly elevated the abundances of *Allobaculum*, *Clostridium*, *Escherichia*, *Enterococcus*, and *Barnesiella* compared to the control, while DHE reversed these alternations (*p* < 0.0001 and *p* < 0.01) (Figure 3D). Meanwhile, DSS down-regulated *Lactobacillus*, *Bifidobacterium*, and *SMB53* abundances compared to the control, which was significantly reversed by DHE administration (*p* < 0.0001 and *p* < 0.01) (Figure 3E). These signs point to the fact that DHE alleviates UC by altering the gut microbial balance.

### 2.5. DHE Modulated DSS-Induced Alterations in Serum Metabolites of Rats

**Figure 4:**
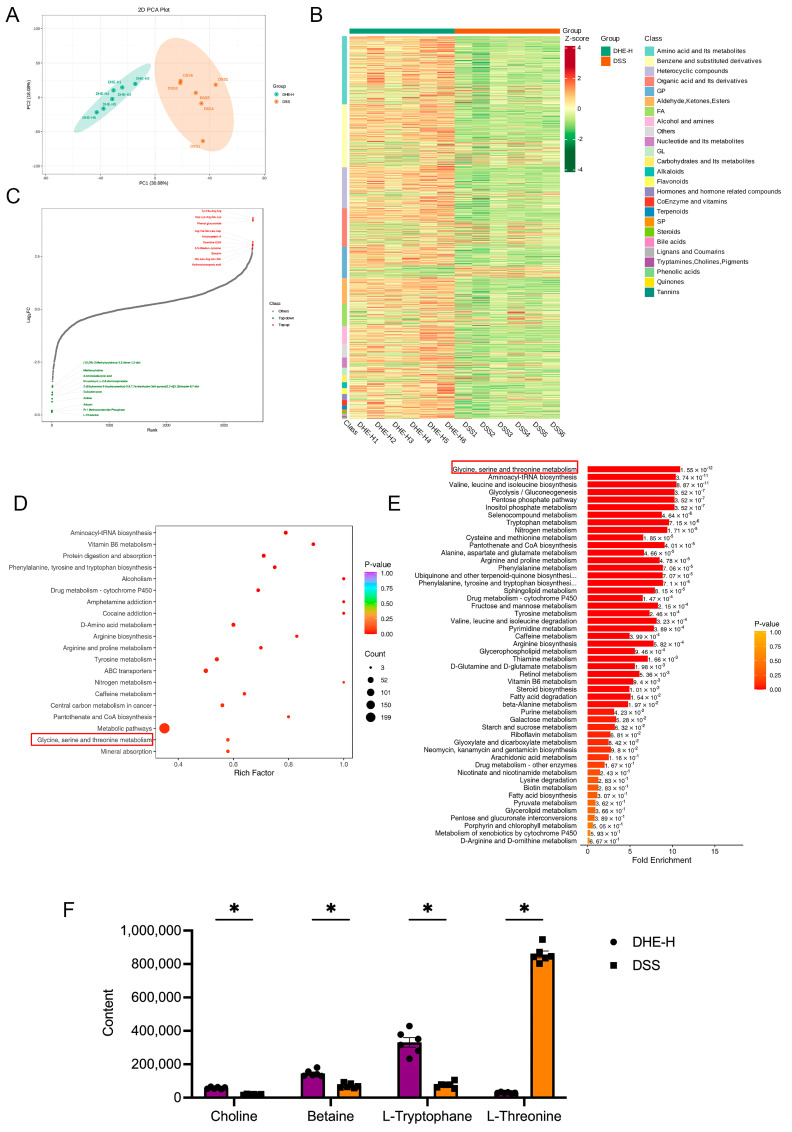
DHE modulated DSS−induced alterations in serum metabolites of rats. (**A**) PCA analysis between the DSS group and DHE−H group. (**B**) The overall clustering diagram between the DSS group and DHE−H group. (**C**) Dynamic distribution of upregulated and downregulated metabolites between the DSS group and DHE−H group. (**D**) KEGG analysis of differential metabolites between the DSS group and DHE−H group. (**E**) MSEA analyses of differential metabolites between the DSS group and DHE−H group. (**F**) Significant metabolites regulated by DHE in glycine, serine, and threonine metabolism (*n* = 6). All data are expressed as mean ± SEM (* *p* < 0.05 indicates statistically significant differences).

To investigate whether DHE intake modulates DSS-induced alterations in serum metabolites, we collected serums from rats in the DHE-H and DSS groups for metabolomic analysis. The principal component analysis (PCA) outcomes showed that the DHE-H and DSS groups shared a well-defined separation (Figure 4A). A total of 1161 differential metabolites were obtained after analyzing 12 rat serum samples, of which 202 metabolites were down-regulated, and 959 metabolites were upregulated after DHE intake, relative to the DSS group. Figure 4B depicts the changes in serum metabolic phenotype after DHE administration. The top 10 metabolites that were upregulated after DHE administration consisted of Tyr-His-Arg-Arg, Asp-Lys-Arg-Glu-Lys, phenol glucuronide, Arg-Val-Ser-Leu-Asp, antanapeptin A, carnitine C3:0, 3,5-diiodo-L-tyrosine, baicalin, Gly-Leu-Arg-Asn-Gin, and hydroxyisocaproic acid (Figure 4C). Meanwhile, the top 10 metabolites that were downregulated after DHE administration were (15,2R)-3-methylcyclohexa-3,5-diene-1,2-diol, methionvIvaline, 2-aminoisobutyric acid, N-succinyl-L, L-2,6-diaminopimelate, 2-(ethylamino)-5-(hydroxymethyl)-5,6,7,7a-tetrahydro-3aH-pyrano[3,2-d][1,3]thiazole-6,7-diol, sulisobenzone, aniline, arbutin, R-1 methanandamide phosphate, and L-threonine (Figure 4C). Moreover, the KEGG analysis suggested that the regulation of DHE in DSS-induced UC rats mainly involved aminoacyl-tRNA biosynthesis, vitamin B6 metabolism, phenylalanine, tyrosine and tryptophan biosynthesis, protein digestion and absorption, and so on (Figure 4D). Furthermore, the metabolic set enrichment analysis (MSEA) outcomes implied that glycine, serine, and threonine metabolism; aminoacyl-tRNA biosynthesis; valine, leucine, and isoleucine biosynthesis; and glycolysis/gluconeogenesis are considered the differences between DSS and DHE-H groups (Figure 4E). Specifically, we focused on the role of DHE in mitigating UC through the modulation of glycine, serine, and threonine metabolism. Specifically, DHE significantly upregulated choline, betaine, and L-tryptophane and down-regulated L-threonine in this metabolic pathway (Figure 4F). Betaine was found to demonstrate a therapeutic role in alleviating UC by suppressing pyroptosis [22]. Collectively, this evidence implies that DHE may alleviate DSS-induced UC by modulating alterations in serum metabolites.

### 2.6. Integrated Network Pharmacology with RNA Sequencing Revealed the Mechanisms of DHE on UC

**Figure 5:**
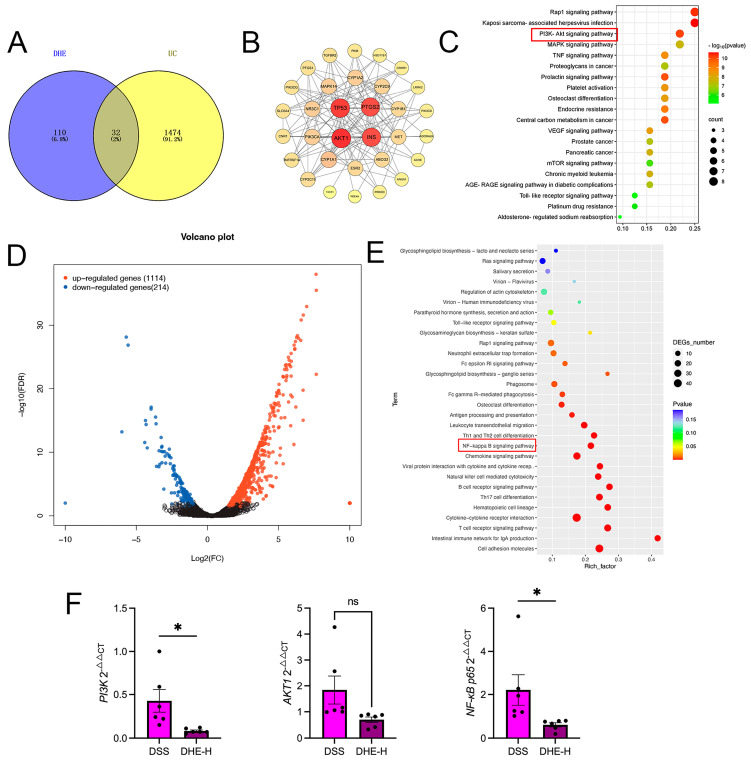
Integrated network pharmacology with RNA sequencing revealed the mechanisms of DHE on UC. (**A**) Venn diagram for targets between DHE and UC by network pharmacology. (**B**) PPI network for core targets. (**C**) KEGG analysis for network pharmacology; Size and color depth of red squares indicate the targets counts and *p*-value. (**D**) The volcano plot of genes in RNA sequencing between the DSS group and DHE−H group. (**E**) KEGG analysis for RNA sequencing between the DSS group and DHE−H group; Size and color depth of red squares indicate the targets counts and *p*-value. (**F**) RT−qPCR for *Pi3k*, *Akt1*, *Nf-κb p65* mRNA (*n* = 3). (* *p* < 0.05 indicates statistically significant differences; ns, no significance).

To unravel the underlying mechanism of DHE UC, we performed network pharmacology. The results of network pharmacology suggested that a total of 32 targets were obtained (Figure 5A). PPI results display that AKT1, PTGS2, TP53, and INS are responsible for the regulation of DHE on UC (Figure 5B). Subsequent KEGG analysis revealed that the PI3K/AKT signaling pathway may be a core regulatory pathway for DHE to improve UC (Figure 5C). In addition, we performed RNA sequencing using rat colon tissues from the DSS and DHE-H groups. The outcomes revealed that DHE administration displayed 1328 significantly altered genes, including 1114 upregulated genes and 214 down-regulated genes, compared with the DSS group (Figure 5D). The results of the subsequent KEGG analysis are shown in Figure 5E. Many lines of evidence have demonstrated that activation of the PI3K/AKT signaling pathway contributes to the activation of the NF-κB signaling pathway in many inflammatory diseases. Therefore, we hypothesized that DHE may alleviate UC by inhibiting the PI3K/AKT/NF-κB signaling pathway. Accordingly, we examined the expression of *Nf-κb p65*, *Akt1*, and *Pi3k* mRNAs in rat colon tissues using RT-qPCR and showed that DHE downregulated the DSS-induced high expression of these genes (*p* < 0.05) (Figure 5E).

### 2.7. DHE-Suppressed DSS Mediated the Activation of PI3K/AKT/NF-κB Signaling Pathway

**Figure 6:**
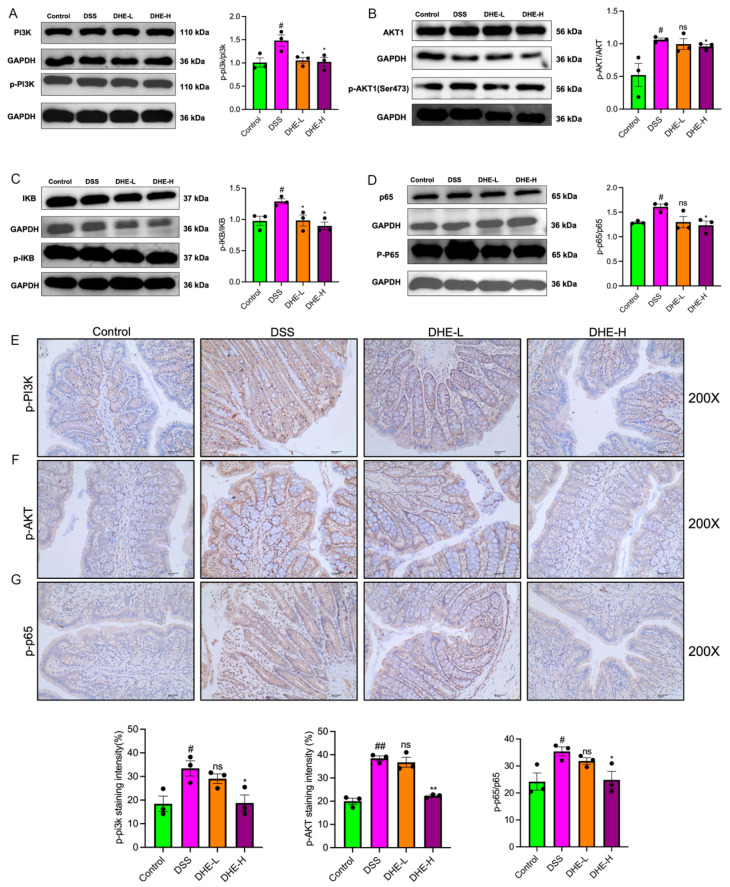
DHE-suppressed DSS mediated the activation of PI3K/AKT/NF-κB signaling pathway. The p-PI3K/PI3K (**A**), p-AKT/AKT (**B**), p-IκB/p-IκB (**C**), p-NF-κB p65/NF-κB p65 (**D**) expression by WB (*n* = 3). The p-PI3K (**E**), p-AKT (**F**), and p-NF-κB p65 (**G**) expression by IHC (*n* = 3). All data are expressed as mean ± SEM (** *p* < 0.01 and * *p* < 0.05 indicate statistically significant differences from the DSS group; ^##^ *p* < 0.01 and ^#^ *p* < 0.01 indicate statistically significant differences from the control group; ns, no significance).

To verify that DHE ameliorates DSS-induced UC by inhibiting the PI3K/AKT/NF-κB signaling pathway, we examined the expression of related proteins using WB. DSS significantly increased the p-PI3K/PI3K ratio and p-AKT/AKT ratio compared with the control group (*p* < 0.05), suggesting the activation of the PI3K/AKT signaling pathway (Figure 6A,B). Meanwhile, DSS intake significantly activated the NF-κB signaling pathway (*p* < 0.05), while DHE intake reversed these changes (*p* < 0.05) (Figure 6C,D). Meanwhile, we detected the positive expression of p-PI3K, p-AKT, and p-NF-κB p65 in rat colon tissues using IHC. Similarly, DSS intake enhanced the positive expression of p-PI3K, p-AKT, and p-NF-κB p65 in rat colon tissues (*p* < 0.05 and *p* < 0.01), whereas DHE administration decreased this trend (*p* < 0.05 and *p* < 0.01) (Figure 6E–G). Collectively, DHE ameliorated DSS-induced UC by inhibiting the PI3K/AKT/NF-κB signaling pathway.

### 2.8. HuProt^TM^20K Proteomics Revealed Direct Binding of DHE to AKT1

**Figure 7:**
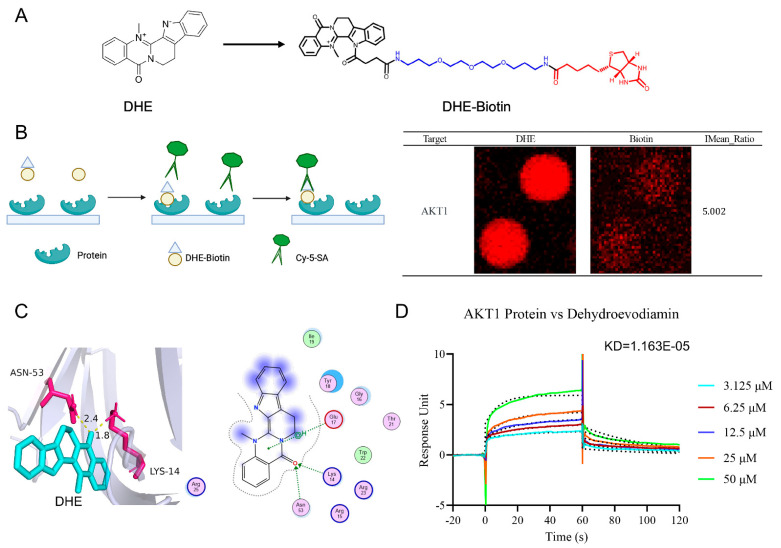
HuProt^TM^20K proteomics revealed direct binding of DHE to AKT1. (**A**) The process of HuProt^TM^20K proteomics. (**B**) The result of HuProt^TM^20K proteomics for AKT1 protein binding to DHE. (**C**) Molecular docking between DHE and AKT1. (**D**) SPR result for AKT1 protein binding to DHE with KD = 1.163 × 10^−5^.

To further resolve the regulatory effects of DHE on the PI3K/AKT/NF-κB signaling pathway, we utilized HuProt^TM^20K proteomics to detect the targets of DHE. We synthesized DHE-biotin as in Figure 7A, and its ^1^H NMR spectrum and mass spectrum are provided in Supplementary Figure S1. HuProt^TM^20K proteomics covers about 20,000 human full-length proteins expressed and purified by eukaryotic expression systems. Upon binding of DHE-biotin to the target protein, it is detected using fluorescent labeling (Figure 7A). A total of 639 targets were obtained, among which AKT1 was detected with an IMean Ratio of 5.002 (Figure 7B). Subsequently, we utilized molecular docking to reveal the mode of interaction between DHE and AKT1. The binding mode of DHE with AKT1 is shown in Figure 7C. DHE was found to bind to ASN-53 and LYS-14 through hydrogen bonding and bind to Glu-17 through pai/H interactions. The affinity of this binding mode is −5.54 KJ/mol. Finally, we detected the affinity between DHE and AKT1 using SPR. DHE had a strong affinity with AKT1 (KD = 1.163 × 10^−5^) (Figure 7D). This evidence suggests that DHE alleviated UC in rats by targeting AKT1 and thereby inhibiting DSS-induced activation of the PI3K/AKT/NF-κB signaling pathway.

## 3. Discussion

As a severe type of IBD, UC severely impairs the quality of survival of the patient community. However, there is a lack of effective drug regimens for long-term administration. As a natural bioactive alkaloid, DHE is beneficial against gastrointestinal diseases. In the current study, we found that DHE was effective in ameliorating symptoms in DSS-induced experimental UC rats by decreasing fecal blood and fecal viscosity. Meanwhile, DHE significantly decreased the levels of MDA, IL-1β, IL-6, and TNF-α and increased the levels of HO-1 and SOD in the intestinal tissues and serum of UC rats, suggesting that it was effective in suppressing inflammatory responses and oxidative stress. In addition, we also confirmed that DHE was able to promote DSS-induced repair of the intestinal epithelial mucosal barrier by increasing the expression of the tight junction proteins ZO-1 and occludin and inhibiting apoptosis. This evidence strongly supports the therapeutic benefit of DHE in DSS-induced experimental UC.

In particular, we analyzed changes in gut microbiology and serum metabolic phenotypes before and after DHE administration. In terms of gut microbial modulation, DHE decreased the abundance of *Allobaculum*, *Clostridium*, *Escherichia*, *Enterococcus*, and *Barnesiella*. *Allobaculum*, a group of bacteria that colonize the inner layer of mucus, are thought to be closely associated with the development of UC [23]. Moreover, recurrent *Clostridium difficile* infection is thought to promote the development of IBD [24]. *Enterococcus faecium* of intestinal origin exacerbated UC by promoting an inflammatory response [25]. Conversely, DHE intake increased the abundance of *Lactobacillus*, *Bifidobacterium*, and *SMB53*. *Lactobacillus johnsonii (L. johnsonii)* could mitigate UC by activating CD206^+^ macrophages and modulating IL-10 emission via the TLR1/2-STAT3 signaling pathway [26]. Similarly, extensive evidence has pointed to *Bifidobacterium longum* as a colonizing bacterium in the gut with therapeutic potential for IBD [27,28]. This evidence implies the potential of DHE to mitigate UC by increasing beneficial flora to reduce harmful flora. In terms of serum metabolite regulation, we focused on the role of DHE in mitigating UC, possibly through the modulation of glycine, serine, and threonine metabolism. Specifically, DHE significantly upregulated choline, betaine, and L-tryptophane in this metabolic pathway. Betaine demonstrated a therapeutic role in alleviating UC [22,29]. This hints at the potential benefit of DHE in alleviating UC by modulating metabolism.

Subsequently, network pharmacology and RNA sequencing implied that DHE alleviated UC by inhibiting the PI3K/AKT/NF-κB signaling pathway. Our results also showed that DHE significantly inhibited the PI3K/AKT/NF-κB signaling pathway in the intestinal tissues of UC rats. Similarly, the upregulation of the PI3K/AKT/NF-κB signaling pathway in UC was confirmed in a large number of studies [30,31,32]. The activated PI3K/AKT signaling pathway mediates the activation of the NF-kB signaling pathway. Phosphorylated AKT activates the NF-kB signaling pathway by phosphorylating IkB and P65. In particular, we revealed that DHE is a novel AKT1 inhibitor using HuProt^TM^20K proteomics and SPR. AKT1 was significantly upregulated in a variety of diseases, suggesting that DHE could be used as an AKT1 inhibitor in other diseases [33,34]. Although this evidence fully supports the therapeutic role of DHE in UC, some limitations need to be mentioned. In terms of modulating gut microbes, future relevant enterobacterial transplants should be performed to conclusively demonstrate the ameliorative effects of DHE. In terms of metabolite regulation, future studies should focus on the role of DHE in the modulation of key enzymes that characterize metabolites. In addition, we found the therapeutic effects of DHE in in vivo experiments, and future studies should be extended to in vitro vectors such as organoids. Finally, although our results support DHE as an AKT1 inhibitor, the specific mode of interaction was not revealed.

## 4. Methodology and Experimental Materials

### 4.1. Chemicals

All chemicals, agents, ELISA kits, and antibodies are shown in Supplementary Table S1.

### 4.2. Establishment of UC Rat Model and Drug Administration Process

A total of 40 SD rats purchased from SPF (Beijing)Biotechnology Co., Ltd. (Beijing, China) were randomized into five groups: control, DSS (dextran sulfate sodium), DHE-L, DHE-H, and 5-ASA. Following five days of adaptive feeding, all groups were given the same volume of 0.9% NaCl fluid for a continuous period of one week. Unlike the control group, the remaining four groups had 4% DSS in the NaCl liquid. In addition, the DHE-L group received 20 mg/kg DHE solution by gavage, the DHE-H group by gavage 40 mg/kg DHE solution, and the 5-ASA group received 300 mg/kg 5-ASA solution by gavage. In the course of this procedure, the daily weight and UC symptoms of every group of rats were documented for pathology scoring analysis. Rules for pathologic scoring refer to the previous study [35]. After one week, all rats were anesthetized and sacrificed, and colon tissues, serum, and intestinal contents of the rats were obtained for subsequent experiments. All rats were housed in SPF-grade animal houses. This study was subject to the Laboratory Animal Center of Chengdu University of Traditional Chinese Medicine (TCM-2022-305).

### 4.3. Measurement of Inflammatory and Oxidative Stress Factors by Enzyme-Linked Immunosorbent Assay (ELISA)

The levels of IL-1β, MDA, IL-6, TNF-α, HO-1, and SOD in rat colon tissue and serum were measured by ELISA, referring to the kit’s description. These kits are all purchased from Quanzhou Ruixin Biotechnology Co., Ltd. (Quanzhou, China). Detailed information of the ELISA kits is displayed in Supplementary Table S1.

### 4.4. Hematoxylin–Eosin (HE) Staining

The obtained rat colon tissues were fixed and sectioned for dewaxing, followed by hematoxylin staining, differentiation, eosin staining, and dehydration sealing. The obtained sections were subjected to microscopy to take pictures.

### 4.5. AB-PAS Staining

Acquired rat colon tissues were fixed, sectioned, dewaxed, and then subjected to PAS oxidation, Schiff’s reagent processing, hematoxylin staining, dehydration, and sealing after differentiation. The slices obtained were subjected to microscopy for photography.

### 4.6. Immunohistochemistry (IHC) Assay

Sodium blocks of rat colon tissue were sectioned for desodiumization, antigen repair, peroxide treatment, and closure, and the primary antibody was incubated at 4 °C overnight. Subsequently, DAB staining, hematoxylin restaining, and dehydration sealing were performed after dropwise addition of secondary antibody. Finally, images were obtained under a microscope. All antibodies are shown in Supplementary Table S1.

### 4.7. RT-qPCR Assay

Rat colon tissues were ground and centrifuged, and 100 μL of chloroform substitute and 15 μL of RNA lysis solution were added for total RNA extraction, followed by detection of RNA concentration. Subsequently, reverse transcription was completed on an ordinary PCR instrument, and amplification was completed on a fluorescent quantitative PCR instrument. Finally, data processing was performed using the ΔΔCT assay. The primer sequences used are shown in Supplementary Table S2.

### 4.8. Western Blotting (WB) Assay

Lysis of rat colon tissue was performed using a RIPA solution dissolved in 100× protease inhibitor and 100× phosphatase inhibitor to obtain a protein solution. Protein denaturation was subsequently performed at high temperature after addition of 5× loading buffer. Protein electrophoresis was performed using SDS-Page. The gel obtained was transferred, closed, and color developed after incubation with primary and secondary antibodies. All antibodies are shown in Supplementary Table S1.

### 4.9. 16S rRNA Sequencing

Fresh intestinal contents were collected for 16S rRNA sequencing. Samples were subjected to DNA extraction, PCR amplification, creation of sequencing libraries, and high-throughput sequencing. The obtained data were denoised or operational taxonomic unit (OUT)-clustered to assess the level of α diversity and detect differential flora.

### 4.10. Metabolomics Assay

The obtained rat serum samples were added to 20% acetonitrile methanol extract, centrifuged, and analyzed by LC-MS/MS. All samples were analyzed under positive and negative ion conditions, same as the previous study [36]. The obtained data were analyzed by PCA, OPLS-DA, and KEGG after library construction and analysis.

### 4.11. Network Pharmacology

The targets of action of DHE were predicted by TCMSP database (https://old.tcmsp-e.com/tcmsp.php) (accessed on 28 June 2024) and Swiss target prediction database (http://www.swisstargetprediction.ch/) (accessed on 28 June 2024). All obtained targets were unified by gene name based on Uniprot database (https://www.uniprot.org/) (accessed on 28 June 2024). The keyword “Ulcerative colitis” was used to search for disease-related targets in the OMIM database (https://omim.org/) (accessed on 28 June 2024) and Gene Cards database (https://www.genecards.org/) (accessed on 28 June 2024), and the obtained targets were screened and removed from the database. The obtained targets were screened and de-replicated. Crossing targets were imported into the STRING database (https://string-db.org) (accessed on 28 June 2024) to obtain the interaction network of the target proteins. Finally, KEGG analysis was performed using the Metascape database (https://metascape.org/) (accessed on 28 June 2024).

### 4.12. RNA Sequencing

Total RNA from rat colon tissue was extracted using TRIzol^®^ Reagent (Invitrogen, Carlsbad, CA, USA), quantified and amplified, and then constructed as a library. The differential genes obtained were subjected to subsequent GO, KEGG, and other analyses.

### 4.13. HuProt^TM^20K Proteomics

HuProt^TM^ proteome microarrays were closed and incubated with DHE-Biotin. After washing, they were incubated with Cy5-Streptavidin. Finally, the data were scanned and recorded using an Axon GenePix 4000B (Axon Instruments, Union City, CA, USA). Protein spots with signal intensity index Z-Score > 2.8 in DHE-Biotin-treated microarrays and those with signal intensity index Z-Score < 2.8 in Biotin-treated microarrays were identified as candidate-positive proteins.

### 4.14. Molecular Docking

The SDF files of the 2D structure and DHE were acquired from the PubChem database, and the 2D structures were converted into MOL2 files of the lowest-energy 3D structures by Chem3D software 2020. PDB files of 3D structures of AKT1 were retrieved using PDB data (https://www.rcsb.org/) (accessed on 28 June 2024), and PDB ID (7myx) was recorded. Autodock Vina software 1.5.7 was used to verify component-disease target interactions. Docking results were visualized using Pymol 2.6.0.

### 4.15. Surface Plasmon Resonance (SPR) Assay

Human AKT1 Protein (10763-H08B; Sino Biological, Beijing, China) was diluted to 40 µg/mL using sodium acetate at pH 4.0, and on experimental channel Fc2, the chip was activated with a mixture of EDC and NHS (1:1) for 420 s (Biacore T200; Cytiva, Marlborough, MA, USA). After the fixation of approximately 11,300 RUs, EA flowed over the surface of the chip to close the excess sites. The reference channel Fc1 was activated and then directly closed. DHE was diluted to 50 µM using Running buffer, and sequential 2-fold gradient dilutions were performed. Analytes flowed through the experimental and reference channels simultaneously, with a binding time of 60 s, a flow rate of 50 µL/min, and a dissociation time of 60 s. Appropriate sequential concentrations (at least 5 concentrations) were selected for Kinetic 1:1 Binding or Steady State Analysis.

### 4.16. Data Analysis

Statistical analysis was carried out with GraphPad Prism 8.0 software. One-way analysis of variance (ANOVA) and nonparametric tests were used to make comparisons between multiple groups, and unpaired *t*-tests were utilized to compare two different groups.

## 5. Conclusions

We demonstrated that DHE effectively alleviated DSS-induced experimental UC and promoted the repair of damaged intestinal epithelial cells by inhibiting inflammation, oxidative stress, and apoptosis regulation. In addition, DHE effectively regulated gut microbes and serum metabolites in UC rats. Mechanistically, DHE exerted therapeutic effects on UC by targeting AKT1 and inhibiting the PI3K/AKT/NF-κB pathway.

## Data Availability

All data are contained within the article.

## Supplementary Materials

The following supporting information can be downloaded at: https://www.mdpi.com/article/10.3390/molecules29174031/s1, Figure S1: 1H NMR spectrum (A) and mass spectrum (B) of DHE-Biotin; Table S1: The information of regents in the study; Table S2: The sequence design of RT-qPCR.
