# Supplementary material for: Dehydroevodiamine Alleviates Ulcerative Colitis by Inhibiting the PI3K/AKT/NF-κB Signaling Pathway via Targeting AKT1 and Regulating Gut Microbes and Serum Metabolism
Source: Molecules. 2024 Aug 26;29(17):4031. doi: 10.3390/molecules29174031 (PMC11397320; doi:10.3390/molecules29174031)
Supplement: Supplementary file 1 [file molecules-29-04031-s001.zip › molecules-3134034-supplementary.pdf]

A B

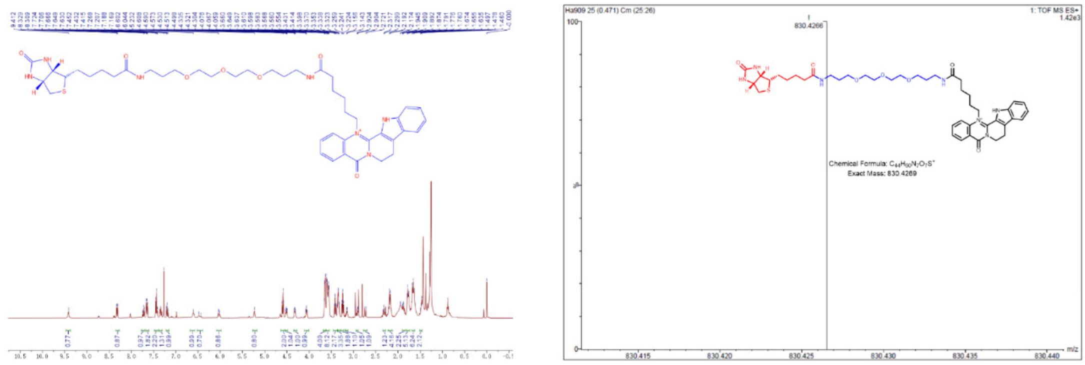

Supplementary figure S1 1H NMR spectrum (A) and mass spectrum (B) of DHE-Biotin.

Supplementary Table S1 The information of regents in the study

Antibody/kit	Source	Identifier
DSS	Chengdu Chroma-Biotechnology Co., Ltd.	CHB-L-237
DHE	ChromaBio	CHB-Q-165
5-ASA	ChromaBio	CHB-M-154
Rat TNF-α ELISA kit	Ruixinbio	RX302058R
Rat IL-16 ELISA kit	Ruixinbio	RX302856R
Rat IL-1β ELISA kit	Ruixinbio	RX302869R
Rat MDA kit	Ruixinbio	RXJ302836R
Rat SOD kit	Ruixinbio	RX301341R
Rat HO-1 kit	Ruixinbio	RX300839R
Anti-AKT1 Antibody	HUABIO	ET1609-47
Anti-p-AKT Antibody	HUABIO	ET1701-36
Anti-ZO-1 Antibody	Servicebio	GB111402
Anti-Pi3k Antibody	HUABIO	ET1609-30
Anti-P-Pi3k Antibody	HUABIO	HA721672
Anti-NF-κB Antibody	HUABIO	ET1603-12
Anti-pNF-κB Antibody	Cell Signaling	310013
Anti-IκBα Antibody	HUABIO	ET1603-6
Anti-pIκBα Antibody	Zenbio	340776
Anti-Occludin Antibody	abcam	ab216327

Supplementary Table S2 The sequence design of RT-qPCR

Primer name	Primer sequence（5'-3')
R-GAPDH-S	CTGGAGAAACCTGCCAAGTATG
R-GAPDH-A	GGTGGAAGAATGGGAGTTGCT
R-NFKB-P65（1）-S	CAGATACCACTAAGACGCACCC
R-NFKB-P65（1）-A	CTCCAGGTCTCGCTTCTTCACA
R-AKT1-S	CGACGTAGCCATTGTGAAGGAG
R-AKT1-A	ATTGTGCCACTGAGAAGTTGTTG
R-Pik3r1-S	CTCCTGGAAGCCATTGAGAAGAA
R-Pik3r1-A	TAAGTCGGCGAGATAGCGTTTG

=
